# Anti-obesity effects by parasitic nematode (*Trichinella spiralis*) total lysates

**DOI:** 10.3389/fcimb.2023.1285584

**Published:** 2024-01-08

**Authors:** Shin Ae Kang, Hak Sun Yu

**Affiliations:** ^1^ Department of Parasitology and Tropical Medicine, School of Medicine, Pusan National University, Yangsan, Republic of Korea; ^2^ Research Institute for Convergence of Biomedical Science and Technology, Pusan National University Yangsan Hospital, Yangsan, Republic of Korea

**Keywords:** obesity, *Trichinella spiralis*, adipocytes, adipogenesis, M1 macrophage

## Abstract

**Background:**

Obesity is an inducible factor for the cause of chronic diseases and is described by an increase in the size and number of adipocytes that differentiate from precursor cells (preadipocytes). Parasitic helminths are the strongest natural trigger of type 2 immune system, and several studies have showed that helminth infections are inversely correlated with metabolic syndromes.

**Methodology/Principal findings:**

To investigate whether helminth-derived molecules have therapeutic effects on high-fat diet (HFD)-induced obesity, we isolated total lysates from *Trichinella spiralis* muscle larvae. We then checked the anti-obesity effect after intraperitoneal administration and intraoral administration of total lysate from *T. spiralis* muscle larvae in a diet-induced obesity model. *T. spiralis* total lysates protect against obesity by inhibiting the proinflammatory response and/or enhancing M2 macrophages. In addition, we determined the effects of total lysates from *T. spiralis* muscle larvae on anti-obesity activities in 3T3-L1 preadipocytes by investigating the expression levels of key adipogenic regulators, including peroxisome proliferator-activated receptor gamma (PPARγ), CCAAT-enhancer-binding protein alpha (C/EBPα) and adipocyte protein 2 (aP2). Oil Red O staining showed that the total lysates from *T. spiralis* muscle larvae decreased the differentiation of 3T3-L1 preadipocytes by decreasing the number of lipid droplets. In addition, the production levels of proinflammatory cytokines IL-1β, IL-6, IFN-γ and TNF-α were examined by enzyme-linked immunosorbent assay (ELISA). *T. spiralis* total lysates decreased intracellular lipid accumulation and suppressed the expression levels of PPARγ, C/EBPα and aP2.

**Conclusion/Significance:**

These results show that *T. spiralis* total lysate significantly suppresses the symptoms of obesity in a diet- induced obesity model and 3T3-L1 cell differentiation and suggest that it has potential for novel anti-obesity therapeutics.

## Introduction

Obesity is a prevalent health problem worldwide and can cause diverse diseases such as cardiovascular disease, type 2 diabetes and cancer (Valenzuela et al., 2023). The prevail of obesity and the number of patients with metabolic disorders induced by obesity are increasing in most countries (Han and Lean, 2016) But, the development of anti-obesity medications progresses and some have been come into market, most of these drugs have been withdrawn because of serious side effects (Scheen et al., 2023). Thus, it is necessary to develop alternative medications to treat obesity without causing adverse effects.

Adipogenesis is the process through which cells differentiate into adipocytes. Pre-adipocytes transform maturing adipocytes with intracellular lipid accumulation (Cao et al., 2023). It is controlled by pivotal adipogenic transcription factors such as CCAAT-enhancer-binding protein alpha (C/EBPα), and peroxisome proliferator activated receptor gamma (PPARγ). C/EBPα and PPARγ also boost adipogenesis by inducing the expression of adipokines including adipocyte fatty acid-binding protein (aP2) (Giby and Ajith, 2014). Since adipocytes play a pivotal role in controlling adipokine secretion, which induces adipogenesis, elucidating the molecular mechanisms that control adipogenesis is important for discovering anti-obesity therapy (Kiernan and MacIver, 2020).

*Trichinella spiralis* is a zoonotic parasite that has broad mammalian host range and is responsible for the disease trichinellosis. *T. spiralis* plays an important role in immune regulation. Parasitic infection drives Th2-biased immune responses and upregulates regulatory T (T_reg_) cells. In a previous study, *T. spiralis* infection activated type 2 immune responses, including M2 macrophages within the adipose tissue, which is important for ameliorating obesity (Kang et al., 2021). *T. spiralis* infection ameliorated glucose and lipid metabolism and reduced lipid accumulation in the liver and adipose tissues. Understandably, instigating a *T. spiralis* infection in the process of developing obesity-improving drugs would be highly dangerous as well as unethical. Therefore, we wanted to check whether there was an obesity-improving effect by isolating parasite-derived molecules (*T. spiralis* total lysates). We also investigated the antiadipogenic effects underlying the action of *T. spiralis* total lysates in 3T3-L1 adipocytes.

## Materials and methods

### Mice

Six-week-old female C57BL/6 mice were purchased from Orient Bio, Inc. (Seongnam, Korea). The mice housed at a specific pathogen-free facility at the Institute for Laboratory Animals of Pusan National University, where all animal studies were conducted. In line with “The Act for the Care and Use of Laboratory Animals” of the Ministry of Food and Drug Safety of Korea, the Pusan National University Animal Care and Use Committee registered and approved the experiments (approval no. PNU- 2021-3149). They were bred in-house at the Pusan National University specific pathogen-free animal facility and accommodated according to regulations. The animals were maintained at a relative humidity of 50–60% and temperature of 25°C ± 2°C under natural light and dark condition (12 h light-dark cycle) with standard diet for 1 week prior to experimentation.

### Study design

**Figure 1:**
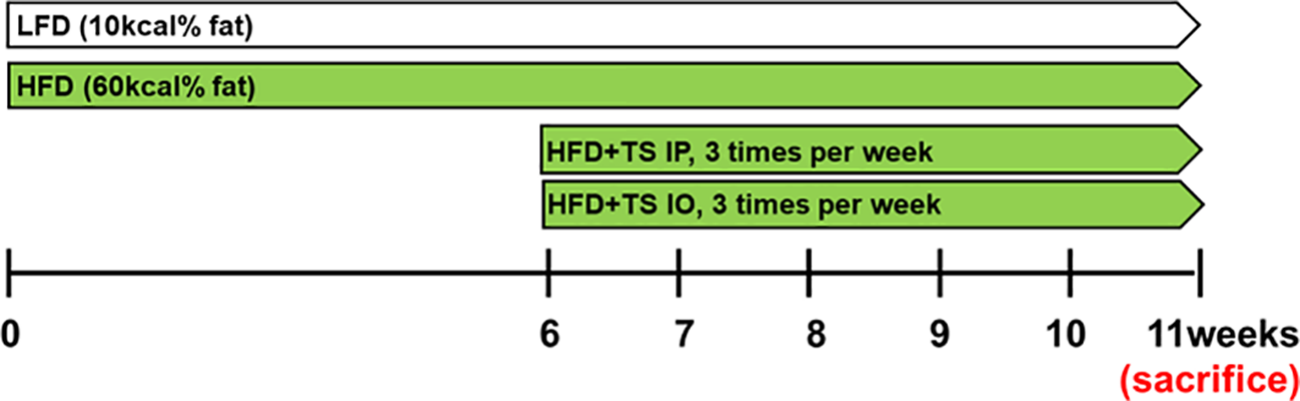
Experimental model. Schematic design illustrates the treatment of *T. spiralis* total lysates in a diet induced obesity model. HFD+TS IP, high-fat diet and intraperitoneal treatment of total lysates; HFD+TS IO, high-fat diet and intraoral treatment of total lysates.

Mice were fed research diets, which were a normal low-fat diet with 10 kcal% fat [LFD], or a high-fat diet with 60kcal% fat [HFD] (New Brunswick, NJ, USA) to induce obesity during 6weeks. We determined the effects of HFD-induced obesity after *T. spiralis* total lysates treatment. After 6 weeks, *T. spiralis* total lysates (50ug) was injected intraperitoneally [HFD+TS IP] or intraorally [HFD+TS IO] once every 3 days for a period of 5 weeks in HFD group. The dietary treatments of LFD and HFD group were maintained throughout the experimental period (**Figure 1**).

### Preparation of *Trichinella spiralis* total lysate

*T. spiralis* was propagated as previously described (Kang et al., 2012). *T. spiralis* larvae were collected as described previously (Kang et al., 2012). *T. spiralis* larvae were homogenized using a disposable Biomasher homogenizer (Nippi, Japan).

### Measurement of body weight and food uptake

Food intake and body weight were weighed weekly, and the food was replaced weekly. The intake was measured by subtracting the remaining food, including any spilled food, from a weighed aliquot every week.

### Measurement of lipids, glucose and insulin in the blood

Plasma total cholesterol, triglyceride, and glucose levels were measured using a Fuji Dri-Chem system (FUJIFILM Corp., Tokyo, Japan). Plasma insulin levels were measured using a mouse insulin enzyme-linked immunosorbent assay (ELISA) kit (SHIBAYAGI Co., Ltd. Gunma, Japan). Plasma insulin levels were determined by ELISA (mouse insulin ELISA, ALPCO, NH, USA).

### Oral glucose tolerance test

In addition, for OGTT, 16-hour fasted mice were administered 20% aqueous glucose solution (fasting body mass (g) × 10 = volume (µL) 20% glucose solution) by gavage through a gastric tube (outer diameter 1.2 mm) that was inserted in the stomach. Blood samples were collected at 0, 15, 30, 60, and 120 min after the administration of glucose. Blood glucose levels were measured using a glucometer (Accu-Chek Active [Model GB] Kit; Roche Diabetes, Mannheim, Germany). The area under the curve (AUC) during OGTT represents glucose levels. To estimate the baseline-corrected AUC, basal glucose levels (time point 0) were taken away all subsequently obtained blood glucose levels for each mouse individually, followed by the calculation of the individual AUCs. Statistical analysis was performed using Student’s two-tailed t-test.

### Measurement of cytokine production

ELISA was used to measure the culture supernatants of the spleen using according to the manufacturer’s instructions (eBioscience, San Diego, CA, USA). Serum concentrations of T helper cell-derived cytokines IL-1β, IL-6 and TNF-α were determined using a ELISA assay (eBioscience, San Diego, CA, USAA).

### Flow cytometry

To investigate macrophage polarization during infection, live cells were isolated from the adipose tissue. The cell surfaces were stained with FITC rat anti-mouse F4/80, PE rat anti-mouse CD11c and APC rat anti-mouse CD206 antibodies (eBioscience) according to the manufacturer’s recommendations. During sample gating, cells were first gated for macrophages. The macrophage gate was used to determine the number of F4/80-positive cells, and CD11c or CD206 surface expression was then determined from the gated population.

### RNA extraction and cDNA synthesis

Gene expression was analyzed as previously described. Briefly, total RNA was isolated using TRIzol Reagent® (Invitrogen, Burlington, ON, Canada). Quantitative reverse transcription-PCR assays were performed by using SYBR™ Select Master Mix (Thermo Fisher Scientific, Waltham, MA, USA) in a QuantStudio 3 Real-Time PCR Instrument (Thermo Fisher Scientific). β- actin was used as a reference gene. The primer sequences are listed in **Table 1.**

**Table 1:** Primers used for realtime PCR.

Primer	sequence
Ap2-F	GATGCCTTTGTGGGAACCT
Ap2-R	CTGTCGTCTGCGGTGATTT
C/EBPα-F	CAAGAACAGCAACGAGTACCG
C/EBPα-R	GTCACTGGTCAACTCCAGCAC
IL-6-F	AGGATACCACTCCCAACAGACC
IL-6-R	AAGTGCATCATCGTTGTTCATACA
Leptin-F	TGGCTTTGGTCCTATCTGTC
Leptin-R	TCCTGGTGACAATGGTCTTG
PPAR γ-F	GGAAGACCACTCGCATTCCTT-
PPAR γ -R	GTAATCAGCAACCATTGGGTCA
Sirt1-F	CGG CTA CCG AGG TCC ATA TAC
Sirt1-R	CAG CTC AGG TGG AGG AAT TGT
TNF-α-F	TACTGAACTTCGGGGTGATTGGTCC
TNF-α-R	CAGCCTTGTCCCTTGAAGAGAACC
β-actin -F	CGTGCGTGACATCAAAGAGAA
β-actin -R	GCTCGTTGCCAATAGTGATGA

### Isolation of adipocytes and stromal vascular fraction from adipose tissue

Gonadal white adipose tissues (gWAT) were collected from infected and uninfected mice, minced, and digested for 1 h at 37°C in HEPES buffer (pH 7.4) containing 0.5 g/L type 1 collagenase from *Clostridium histolyticum* (Sigma-Aldrich, St. Louis, MO, USA) and 2% (w/v) dialyzed bovine serum albumin (Fraction V; Sigma-Aldrich). Disaggregated adipose tissue was filtered through a 100 mm nylon mesh. The residue of the gonadal adipose tissue homogenate was used to isolate the SVF cells for flow cytometry. The sample was centrifuged for 10 min at 350 g, the supernatant was removed and the pellet was treated with ACK lysis buffer (Fujisaka et al., 2009).

### Cell culture

Cells from SVF (5x10^6^ cells/mL) were cultured in 5% CO2 incubator at 37°C for 24 hours in the presence or absence of Ionomycin (5 mg/mL) and PMA (0.4 mg/mL). After incubation, the supernatant was collected and stored at -80°C until use. Cytokine production were determined by ELISA.

### 3T3-L1 cell differentiation

Mouse 3T3-L1 preadipocytes were purchased from ATCC, Manassas, VI, USA. 3T3-L1 cells were cultured in Dulbecco’s modified Eagle’s medium (DMEM) (Thermo Fisher Scientific) containing 10% bovine calf serum (ATCC) and 1% penicillin/streptomycin (Thermo Fisher Scientific) and maintained at 37°C in a humidified atmosphere with 5% CO2. Cells were differentiated 2 days after confluence, and then were stimulated with DMEM (4.5 g/L glucose) containing 10% fetal bovine serum (FBS) (Corning, Corning, NY, USA), 1 g/mL insulin, 0.5 mM isobutylmethylxanthine (IBMX), 1 μM dexamethasone, and 1, 5, or 10 µg of total lysate, or PBS (for control cells) for 2 days. On day 2, the differentiation medium was replaced with DMEM (4.5 g/L glucose) containing 10% FBS, 1 μg/mL insulin, and changed every two days from days two to ten.

### Oil red O staining

The Oil Red O staining protocol was modified from that described by *Pacifici* et al. (Pacifici et al., 2020). The differentiated cells were washed with phosphate-buffered saline (PBS, pH 7.4, Sigma-Aldrich) and fixed in 4% formalin (Sigma-Aldrich) for 20-30 min at room temperature. The cells were then washed with double-distilled water and incubated with 60% isopropanol for 5 min at room temperature (Sigma-Aldrich). After that, the cells were stained with Oil Red O solution (0.5 g/L, Sigma Aldrich) for 30 min at room temperature and washed completely with double-distilled water. Images were acquired using an optical microscope. Oil Red O dye retained in the cells was eluted in pure isopropanol and quantified at 490 nm using a microplate reader.

### Western blot analysis

3T3-L1 adipocytes were lysed using PRO PREP™ Protein Extraction Solution (iNtRON Biotechnology, Seangnam, Korea). Proteins (30 µg) were separated on 10% sodium dodecyl sulfate polyacrylamide gels (SDS-PAGEs). Next, proteins were transferred to nitrocellulose membranes, blocked in 5% skim milk in TBST (10 mM Tris pH 8.0, 150 mM NaCl, and 0.05% Tween 20) for 1 h, and incubated overnight with primary antibodies against PPARγ, C/EBPα and actin (Cell Signaling Technology, Danvers, MA, USA) at 4°C. After washing with TBST, the membranes were incubated with horseradish peroxidase-conjugated secondary antibodies at room temperature for 1 h. Proteins were detected using an enhanced chemiluminescence reagent (GE Healthcare, Chicago, IL, USA).

### Statistical analysis

Statistical analysis was performed using GraphPad Prism software (version 5.0; GraphPad, Inc., La Jolla, CA, USA), with Student’s t-test or analysis of variance (ANOVA). Multivariate data analysis and group differences were assessed using one-way or two-way ANOVA, followed by Bonferroni’s post-hoc test. All experiments were conducted in triplicates and showed similar results. The results are expressed as the mean ± SD (standard deviation) from each experiment.

## Results

### *Trichinella spiralis* total lysates treatment reduces obesity in HFD-fed obese mice

**Figure 2:**
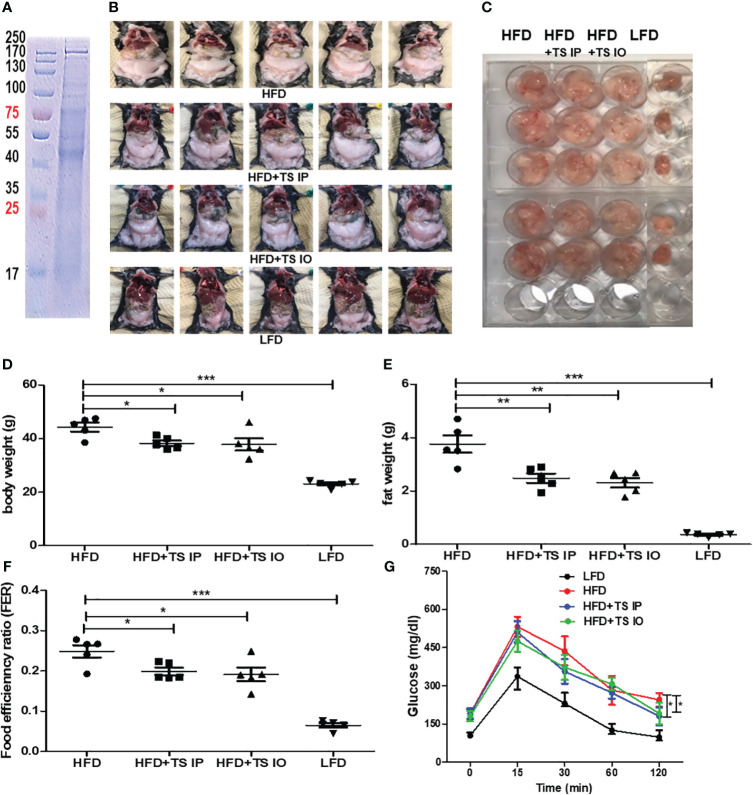
The effect of *T. spiralis* total lysates on body weight and food efficiency ratio (FER) in high-fat diet (HFD)-fed mice. Total lysates were isolated *T. spiralis* muscle larvae. **(A)** Sodium dodecyl sulfate polyacrylamide gel electrophoresis (SDS-PAGE) of total lysates from *T. spiralis* muscle larvae. Obesity was induced in mice by feeding them a HFD for six weeks. The mice were then intraperitoneally injected with *T. spiralis* total lysate three times per week for five weeks. **(B)** Images of representative mice from each group (LFD, HFD, HFD+TS IP, and HFD+TS IO) after five weeks. **(C)** Representative image of gonadal adipose tissue. **(D, E)** Body weight and gonadal fat. **(F)** Food efficiency ratio (FER) = increased body weight (g)/food intake (g). **(G)** Overnight-fasted mice were administered 20% glucose by gastric gavage. Blood samples were collected at 0, 15, 30, 60, 90, and 120 min. Blood glucose levels measured using a glucometer. (*; *p < 0. 05*, **; *p < 0.01* ***; *p < 0.001*, n = 5 mice per group, three independent experiments). LFD, low-fat diet; HFD, high-fat diet; HFD+TS IP, high-fat diet and intraperitoneal treatment of total lysates; HFD+TS IO, high-fat diet and intraoral treatment of total lysates.

To determine the effect of *T. spiralis* total lysates on HFD-induced obesity, C57BL/6 female mice were fed a HFD for 6 weeks to induce obesity. *T. spiralis* total lysate was administered either intraorally or intraperitoneally three times a week for five weeks (**Figure 1**). All the groups were assayed for body weight, food efficiency ratio [FER= total body weight gain (g)/total food intake (g)], final fat mass, and blood biochemical parameters. **Figure 2A** shows a protein electrophoresis image of *T. spiralis* total lysates. Several bands over 40 kDa were detected, but no single bands. **Figures 2B, C** show photographs of representative mice from each group (LFD, HFD, HFD+TS IP, and HFD+TS IO) after six weeks. As expected, HFD feeding lead to a significant increase in body weight and fat mass in the total lysate treatment group. However, significant differences were observed in body and fat weight of mice treated with *T. spiralis* total lysates (HFD+TS IP and HFD+TS IO) and non-treated HFD-fed mice (HFD) (*p < 0.005) (**Figures 2D, E**). According to the food efficiency ratio (FER) equation, a change in body weight was the most important factor influencing the FER, as there were no significant changes in the food intake of the various groups of mice. Thus, it is possible to apply the FER as an indicator; a small FER value can denote obesity prevention. In our experiments, food intake did not differ significantly between *T. spiralis* total lysate-treated and non-treated HFD-fed mice. FER was significantly higher in the HFD-fed mice than in the LFD-fed mice (***p < 0.001). *T. spiralis* total lysate-treated HFD-fed mice had significantly lower FER than non-treated HFD-fed mice (*p < 0.005) (**Figure 2F**).

### *Trichinella spiralis* total lysates treatment could ameliorate glucose and lipid metabolism

**Figure 3:**
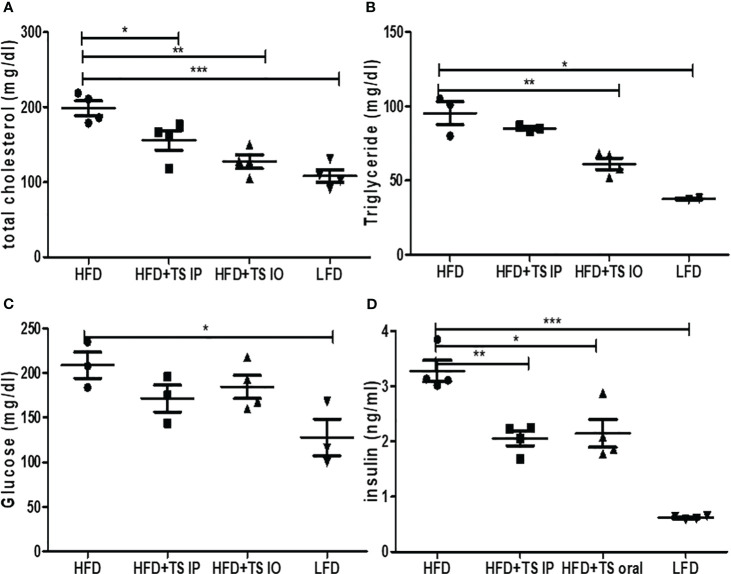
The Effect of *T. spiralis* total lysates on serum lipid profiles, glucose and insulin levels of mice fed an HFD. **(A)** Serum total cholesterol. **(B)** Serum triglyceride. **(C)** Serum glucose. **(D)** Serum insulin. To evaluate the effects of *T. spiralis* total lysates on serum lipid profiles, glucose and insulin. The serum biochemical parameters were assessed in mice of each group (LFD, HFD, HFD+IP, HFD+IO). (*; *p < 0.05*, **; *p < 0.01*, ***; *p < 0.001*, n = 5 mice/group, three independent experiments) LFD, low-fat diet; HFD, high-fat diet; HFD+TS IP, high-fat diet and intraperitoneal treatment of total lysates; HFD+TS IO, high-fat diet and intraoral treatment of total lysates.

Obesity induces abnormal glucose and lipid metabolism. To assess glucose metabolism, we examined whether *T. spiralis*-mediated attenuation of obesity improved the harmful effects of obesity. In a previous study, infection of *T. spiralis* ameliorated glucose and lipid metabolism. In all groups of mice, maximum plasma glucose levels were reached 15 min after the glucose challenge. In contrast, few glucose eliminations were monitored between 15 and 30 min in high-fat diet–fed mice, suggesting severe glucose intolerance. Glucose tolerance tests showed that when HFD mice were challenged with glucose, they had higher blood glucose levels, whereas *T. spiralis* total lysate-treated mice showed improved glucose tolerance at various time points in the tolerance test. The 60- and 120-minute insulin response to the intraorally glucose challenge were significantly weaken after high-fat feeding compared with those in *T. spiralis* total lysate-treated mice (*p < 0.001) (**Figure 2G**). *T. spiralis* total lysates-treated mice showed significantly decreased total cholesterol (**Figure 3A**), triglyceride (**Figure 3B**) and insulin (**Figure 3D**) (*p < 0.005) levels in HFD-fed mice, however this difference was not significant for glucose levels (**Figure 3C**).

### Treatment of *Trichinella spiralis* affected immune response in the HFD-fed obese mice

**Figure 4:**
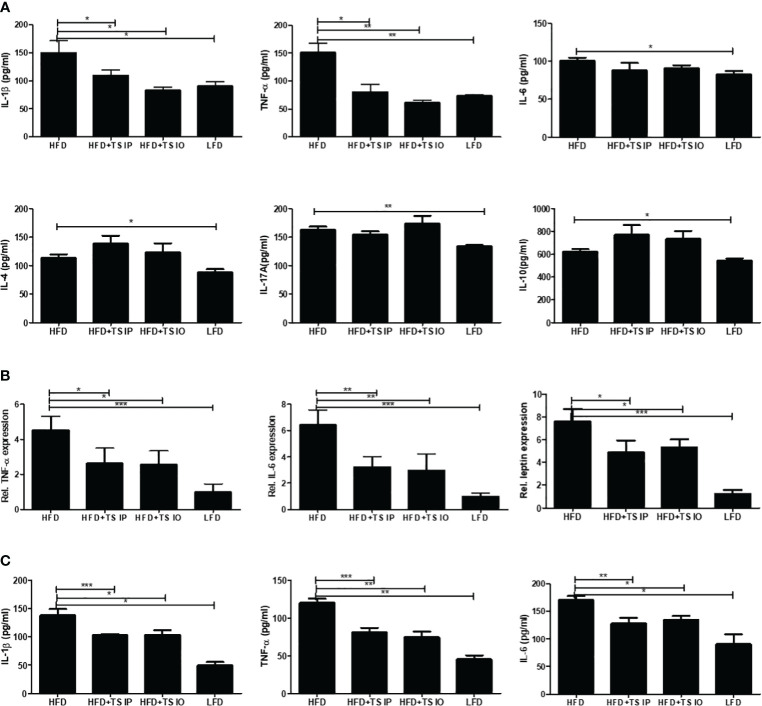
The production of cytokines in the spleen and the m-RNA expression levels in white adipose tissue. **(A)** Cytokine production was measured in lymphocytes isolated from the mouse spleen. Lymphocytes were activated using anti-CD3 antibody. The wells were incubated with 0.5 mg/mL of anti-CD3 for 16 h at 4 °C, and then the lymphocytes isolated from the spleen were added to the well and incubated for 3 days. After activation, the cytokine concentrations in the supernatant were measured using ELISA kits in accordance with the manufacturer’s instructions. **(B)** RNA isolated form gonadal white adipose tissue. Relative mRNA expression confirmed the expression of adipokine genes. The results show that *T. spiralis* total lysates treatment clearly decreased the expression of IL-6, leptin and TNF-α, as well as the mRNA of their target genes, including aP2. **(C)** Serum concentrations of T helper cell-derived cytokines IL-1β, IL-6 and TNF-α were determined using using ELISA kits in accordance with the manufacturer’s instructions. HFD induction increases the secretion of systemic cytokines into the serum. *T. spiralis* total lysates treatment significantly decreased the expression of IL-1β, IL-6 and TNF-α. Bars represent the means ± SD from three independent experiments. (*; p < 0. 05, ** p < 0.01 *** p < 0.001, n = 5 mice/group, three independent experiments) LFD, low-fat diet; HFD, high-fat diet; HFD+TS IP, high-fat diet and intraperitoneal treatment of total lysates; HFD+TS IO, high-fat diet and intraoral treatment of total lysates.

To evaluate the effects of *T. spiralis* treatment on cytokine profiles, ELISA was conducted for IL-1β, L-6, TNF-α, IL-4, IL-17A, and IL-10. **Figure 4A** shows the levels of the six cytokines in the spleen. As expected, the HFD group mice had higher proinflammatory cytokines such as TNF-α, IL-1β and IL-6. Moreover, *T. spiralis* total lysates-treated mice (HFD+TS IP, HFD+TS IO) had significantly decreased proinflammatory cytokines, such as IL-1β and TNF-α, compared to the HFD group (*p < 0.001). In contrast, the production of the Th2 cytokine (IL-4), T_reg_-related cytokines (IL-10) and Th17 cytokines (IL-17A) was not significantly increased compared to that in the HFD group. We check the m-RNA expression level of adipokines including IL-6, TNF-α and leptin in gonadal white adipose tissue (**Figure 4B**). The results showed that adipokines including IL-6, TNF-α and leptin are significantly increased due to diet- induced obesity, but the increased adipokines are significantly decreased due to *T. spiralis* total lysates treatment.

We used an ELISA assay to measure the levels of T helper cell-derived cytokines IL-1β, IL-6 and TNF-α in serum (**Figure 4C**). HFD mice exhibit increased levels of systemic (IL-1β, IL-6 and TNF-α) inflammatory cytokines. We detected a significant decrease in immune and AT-mediated cytokines in the group of mice fed HFD compared to those treated *T. spiralis* total lysates. These data suggest that *T. spiralis* total lysates downregulate the chronic inflammation, at least in part decreasing circulating levels of systemic cytokines. These results suggest an evident impact of parasite-derived molecule treatment on cytokine production profiles and effector T cell differentiation in HFD-fed mice by suppressing Th1 cell responses.

### Treatment of *Trichinella spiralis* induces the ‘anti-inflammatory’ M2 levels by a balance transit in M1/M2 macrophage ratio

**Figure 5:**
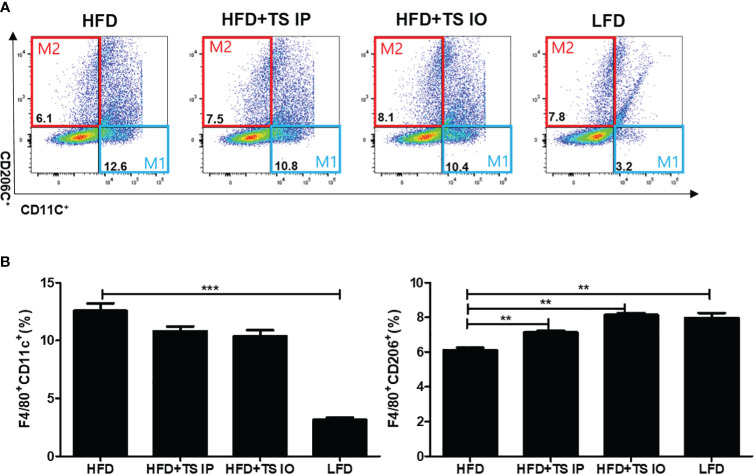
Expression of M1 adipose tissue macrophages (F40/80+CD11c+) and M2 adipose tissue macrophages (F40/80+CD206c+). To evaluate the population of macrophages after the administration of *T. spiralis* total lysate (IO and intraperitoneal injections), we evaluated the adipose tissue macrophage marker (CD11c+, CD206+) levels in the adipose tissue macrophages of mice. Gonadal adipose tissue from mice was digested with collagenase, and adipose tissue macrophages were isolated. Cells were stained with FITC rat anti-mouse F4/80, PE rat anti-mouse CD11c, and APC rat anti-mouse CD206 antibodies. **(A)** After staining, the plots indicate the expression levels of the proinflammatory M1 adipose tissue macrophage marker (CD11c+) and M2 adipose tissue macrophage marker (CD206+) in gated F4/80 positive cells. **(B)**. (** p < 0.01, *** p < 0.05, n = 5 mice/group, three independent experiments). LFD, low-fat diet; HFD, high-fat diet; HFD+TS IP, high-fat diet and intraperitoneal treatment of total lysates; HFD+TS IO, high-fat diet and intraoral treatment of total lysates.

**Figure 6:**
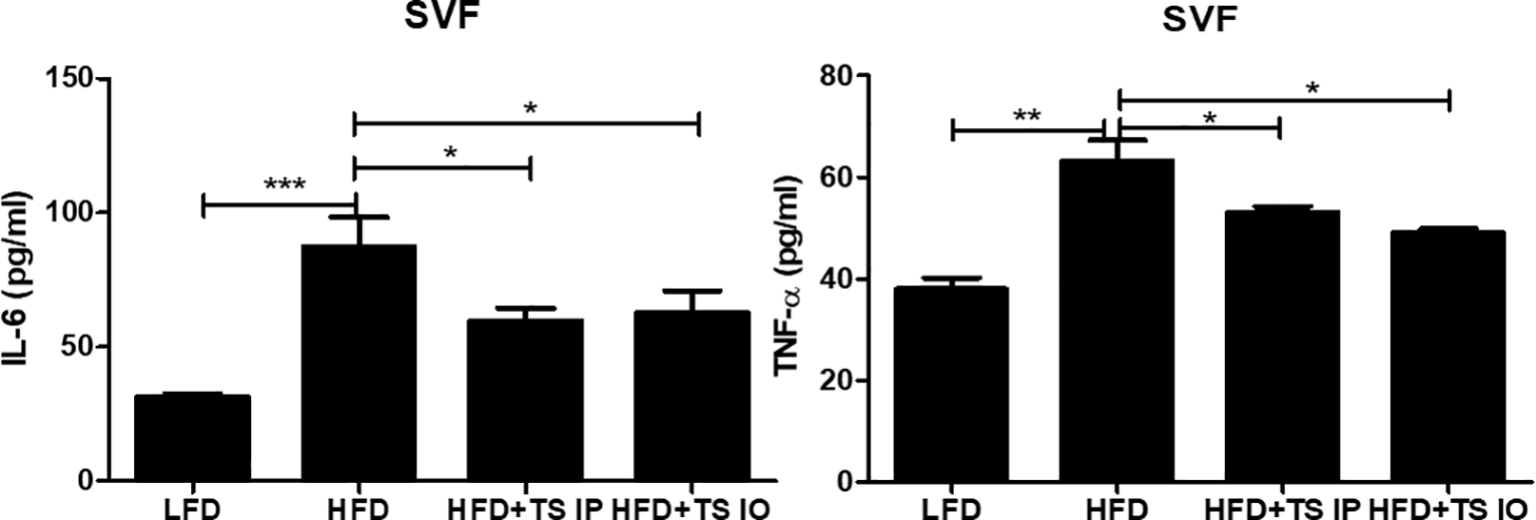
Cytokine production of in the SVFs. Cytokine production was measured in lymphocytes isolated from the stromal vascular fraction of the gonadal white adipose tissue (gWAT). Cells from SVF (5x106 cells/mL) were cultured in 5% CO2 incubator at 37°C for 24 hours in the presence or absence of Ionomycin (5 mg/mL) and PMA (0.4 mg/mL). After incubation, the supernatant was collected and stored at -80°C until use. Cytokine production were determined by ELISA. (*; p < 0. 05, ** p < 0.01 *** p < 0.001, n = 5 mice/group, three independent experiments). LFD, low-fat diet; HFD, high-fat diet; HFD+TS IP, high-fat diet and intraperitoneal treatment of total lysates; HFD+TS IO, high-fat diet and intraoral treatment of total lysates.

Obesity is related to macrophage accumulation in the adipose tissue. The number and population of macrophages in adipose tissue differs according to the metabolic state. We assessed whether helminth-derived molecules induced an imbalance in the ratio of M1/M2 macrophages in gonadal fat tissue. The results from our FACS analysis showed that HFD feeding of untreated obese mice led to an increase in M1 cells (F4/80^+^ CD11C^+^) and this increase was prevented by treatment with *T. spiralis* total lysate (***p < 0.01) (**Figures 5A, B**). In contrast, HFD feeding of non-treated obese mice lead to decreased M2 cells (F4/80^+^ CD206^+^) and *T. spiralis* total lysate treatment group (HFD+TS IP, HFD+TS IO) induced increase of M2 macrophages (F4/80^+^ CD206^+^) in the gonadal fat tissue (**p < 0.05). These results suggest that treatment with *T. spiralis* total lysate may protect against diet-induced obesity by promoting macrophage polarization to anti-inflammatory M2 macrophages in the gonadal fat of obese mice. Adipose tissues also produce cytokines known as adipokines that are involved in the development of metabolic diseases (Hotamisligil, 2017). Because of the importance of these cytokines in metabolism, we analyzed their production in gWAT. SVF cells were isolated from the adipocytes, cultured for 24 h, and cytokine levels were measured in the supernatants. We found no difference in the production of Th2 cytokines (IL-4), Th17 cytokine (IL-17A) and anti-inflammatory cytokine (TGF-β) by SVF cells when comparing HFD and *T. spiralis* total lysates treatment group (data not shown). On the other hand, the production of proinflammatory adipocytokines like IL-6 and TNF-α, by SVF cells, was increased in the HFD group when compared to *T. spiralis* total lysates treatment group (HFD+TS IP, HFD+TS IO) (**Figure 6**). These data suggest that the effect of *T. spiralis* total lysates treatment on metabolic parameters might be associated with a reduction in pro-inflammatory cytokines rather than the production of anti-inflammatory or homeostatic adipocytokines.

### Liver histopathology

**Figure 7:**
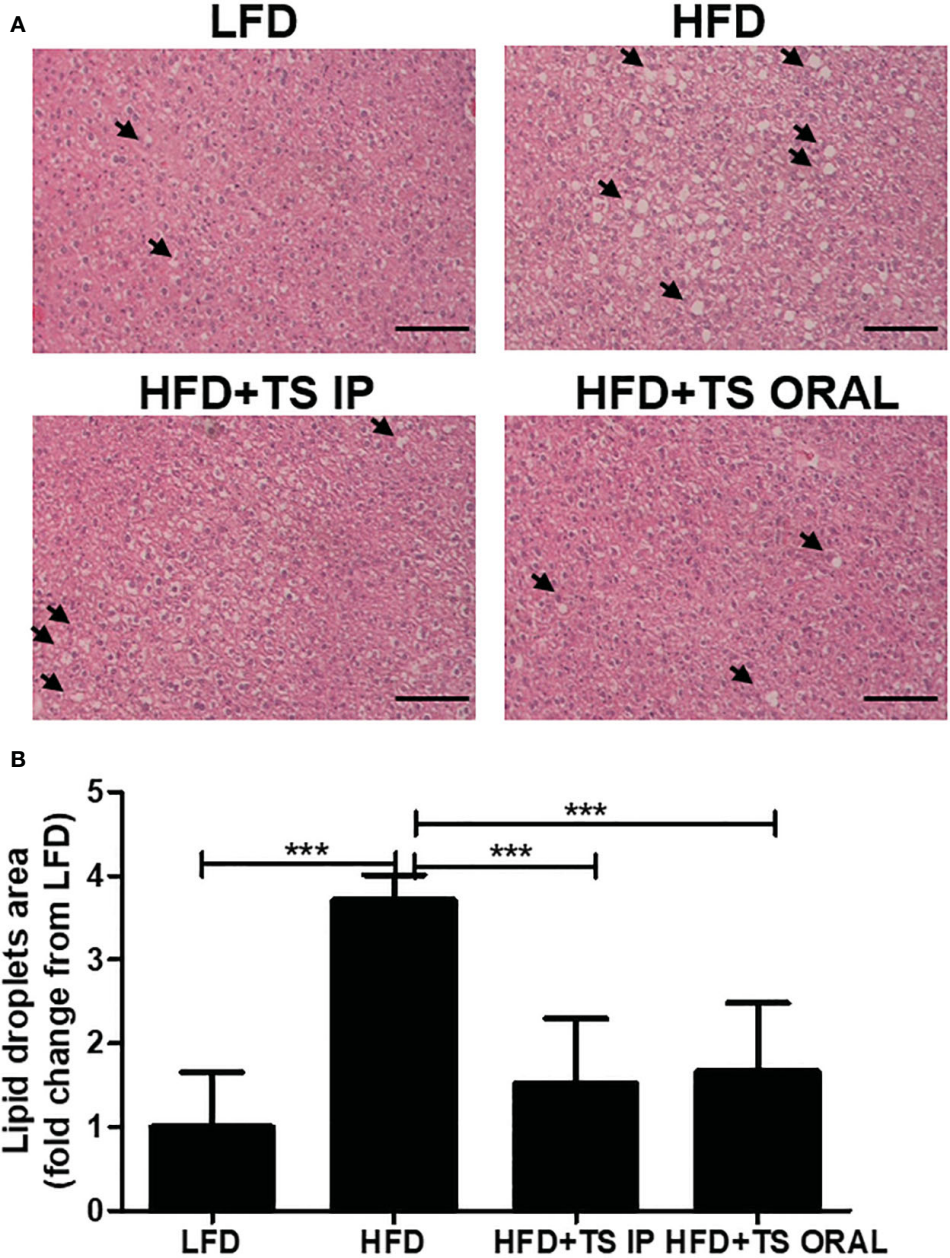
Histopathological alterations in the liver of mice from each group. Histopathological alterations in the livers of mice in each group. Hematoxylin and eosin (H&E) staining of the liver (200× magnification). Arrows indicate lipid droplets. Scale bar = 50 µm. **(A)** Representative images of one section per mouse are shown **(B)**. Area of lipid droplets was quantified by microscopy (***p < 0.001, n = five mice/group, in three independent experiments). LFD, low-fat diet; HFD, high-fat diet; HFD+TS IP, high-fat diet and intraperitoneal treatment of total lysates; HFD+TS IO, high-fat diet and intraoral treatment of total lysates.

Histopathological analysis of the liver was assessed by hematoxylin and eosin (H&E) staining (**Figure 7A**). Hepatic H&E staining revealed that HFD increased hepatocellular vacuolation and the frequency of lipid droplets. Especially, these histopathological changes were attenuated by *T. spiralis* total lysate (**Figure 7B**). Hepatic H&E staining showed that the high fat diet increased hepatocellular vacuolation due to cellular lipid accumulation, enlarged hepatocytes, shifted nuclei toward the plasma membrane, and narrowed sinusoidal channels. In summary, we suggest that treatment with *T. spiralis* total lysate could regulate diet-induced lipid accumulation in white adipose tissue and the liver, which could reduce the risk of fatty liver disease and excessive adipocyte lipid accumulation, which could induce severe hepatic steatosis.

### *Trichinella spiralis* total lysates regulates 3T3-L1 differentiation

**Figure 8:**
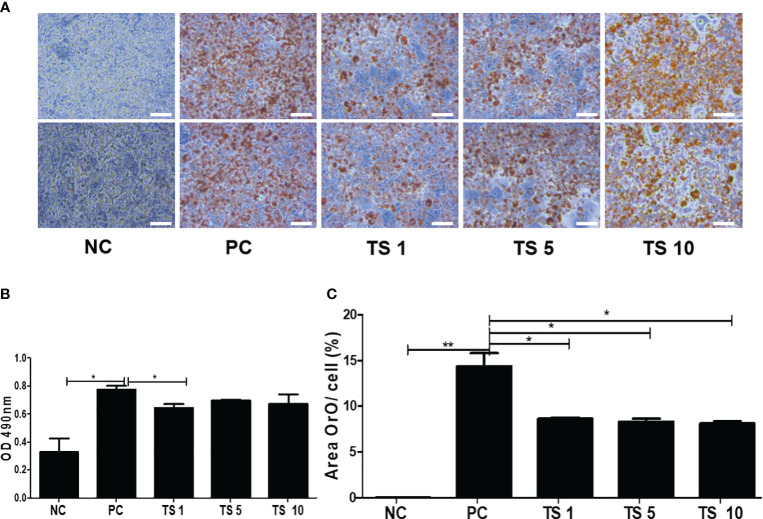
The effects of *T. spiralis* total lysates on adipocyte differentiation of 3T3-L1 cells. Preadipocyte 3T3-L1 cells were induced to differentiate by the addition of MDI (IBMX, DEX, and insulin). 3T3-L1 cells were treated with 1, 5, or 10 μg *T. spiralis* total lysates during differentiation. **(A)** The effect of *T. spiralis* total lysates on lipid accumulation in 3T3-L1 adipocytes. Oil Red O staining images of 3T3-L1 adipocytes at the terminal stage of differentiation. **(B)** Oil Red O was extracted from cells with 100% isopropanol and absorbance was determined spectrophotometrically at 490 nm. **(C)** 3T3-L1 adipocytes positive for Oil Red O staining were quantified. Scale bars represent 100 μm. (*; p < 0. 05, **; p < 0.01, n = 4 samples per group, three independent experiments). NC, negative control, undifferentiation; PC, positive control, differentiation; TS 1, treatment of *T. spiralis* total lysates 1 µg; TS 5, treatment of *T. spiralis* total lysates 5 µg; TS 10, treatment of *T. spiralis* total lysates 10 µg.

To investigate whether *T. spiralis* total lysates regulate adipocyte maturation, the differentiation of 3T3-L1 cells was induced, and the lipids intracellular storage was monitored by performing Oil Red O staining on day 10. Treatment with *T. spiralis* total lysates significantly reduced lipid accumulation at 1, 5, 10 μg, compared with control cells (**Figure 8A**). It was confirmed by measuring the absorbance of Oil Red O at 490 nm. Lipid accumulation in 3T3-L1 cells was significantly decreased by 35% following *T. spiralis* total lysates, compared to the control (p < 0.01) (**Figures 8B, C**). Taken together, these results highlight the related ability of *T. spiralis* total lysate in regulating adipocyte differentiation, which may highly contribute to reduced adipose cell mass accumulation.

### *Trichinella spiralis* total lysates exerts anti-inflammatory effects

**Figure 9:**
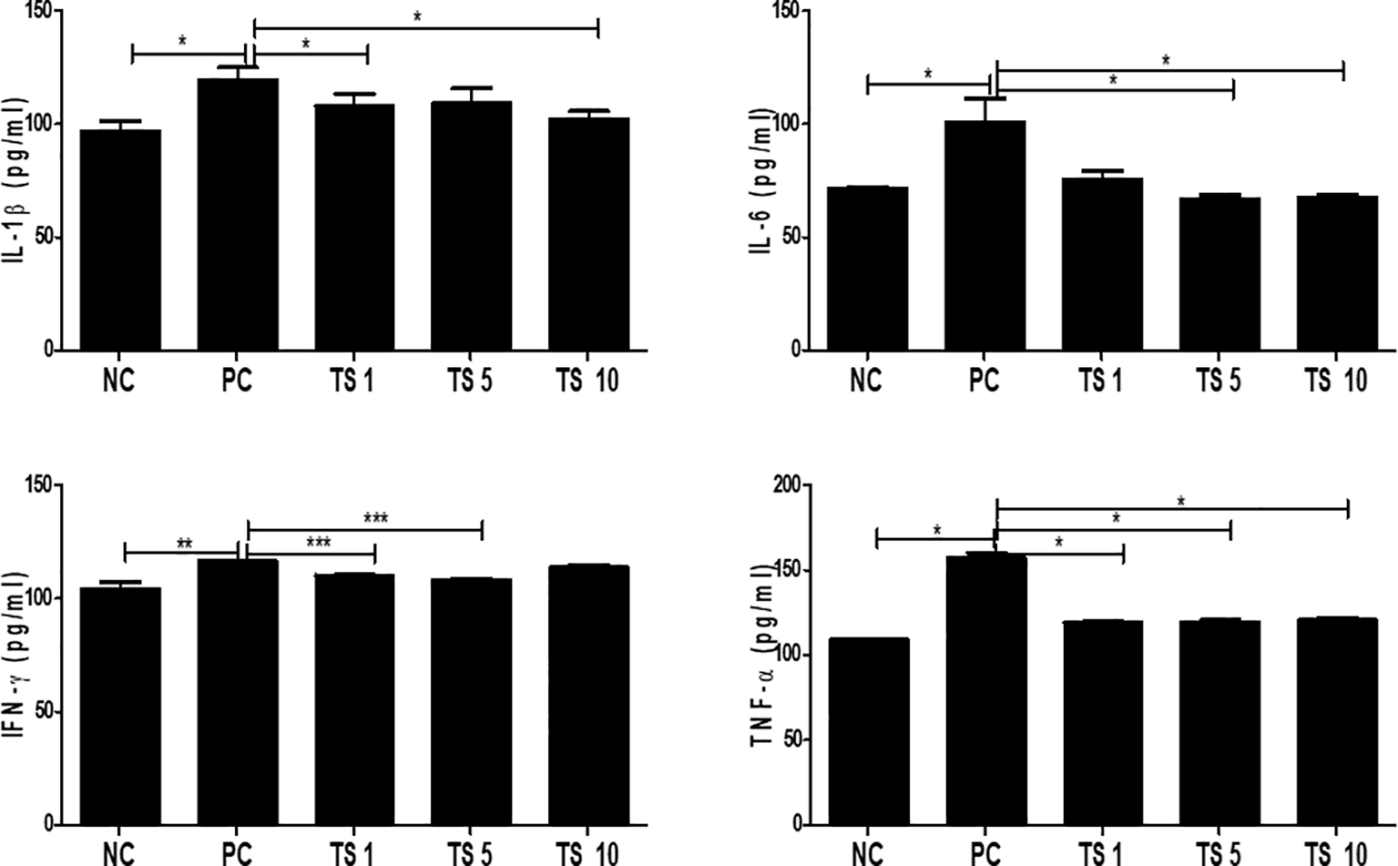
Effects of *T. spiralis* total lysates on the levels of IL-1β, IL-6, IFN-γ, and TNF-α in 3T3-L1 Adipocytes. Cytokines were detected in culture supernatants by ELISA. The cytokine concentrations in the supernatants of 3T3L1 cells were measured using ELISA kits in accordance with the manufacturer’s instructions. Values are expressed as mean ± SD. As expected, the differentiation group had higher proinflammatory cytokine such as IL-1β, IL-6, INF-γ and TNF-compared to non-differentiation group. Treatment of *T. spiralis* total lysates were significantly reduced proinflammatory cytokine such as IL-1β, IL-6, INF-γ and TNF-(*; p < 0. 05, ** p < 0.01, *** p < 0.001, n = 4 samples per group, three independent experiments). NC, negative control, undifferentiation; PC, positive control, differentiation; TS 1, treatment of *T. spiralis* total lysates 1 µg; TS 5, treatment of *T. spiralis* total lysates 5 µg; TS 10, treatment of *T. spiralis* total lysates 10 µg.

Adipogenesis is involved in enhancing the proinflammatory state (Okuno et al., 2018). We evaluated whether *T. spiralis* total lysate reduced the proinflammatory state typically of adipocyte differentiation. In particular, we detected the levels of proinflammatory cytokine such as IL-1β, IL-6, IFN-γ, and TNF-α in supernatants of fully differentiated adipocytes treated with *T. spiralis* total lysates. Although they did not decrease in a concentration-dependent manner, all cytokine (IL-1β, IL-6, IFN-γ, TNF-α) levels were significantly reduced by *T. spiralis* total lysates (p < 0.01) compared to those in control cells. We found no difference in the production of other cytokines (IL-4, IL-10, IL-17A) when comparing differentiation and *T. spiralis* total lysates treatment group (data not shown) (**Figure 9**). *T. spiralis* total lysates may contribute to enhancing the changes in obesity-induced systemic conditions through their anti-inflammatory effect.

### *Trichinella spiralis* total lysates weaken adipogenesis by controlling the C/EBPα and Sirt1/PPARγ pathway

**Figure 10:**
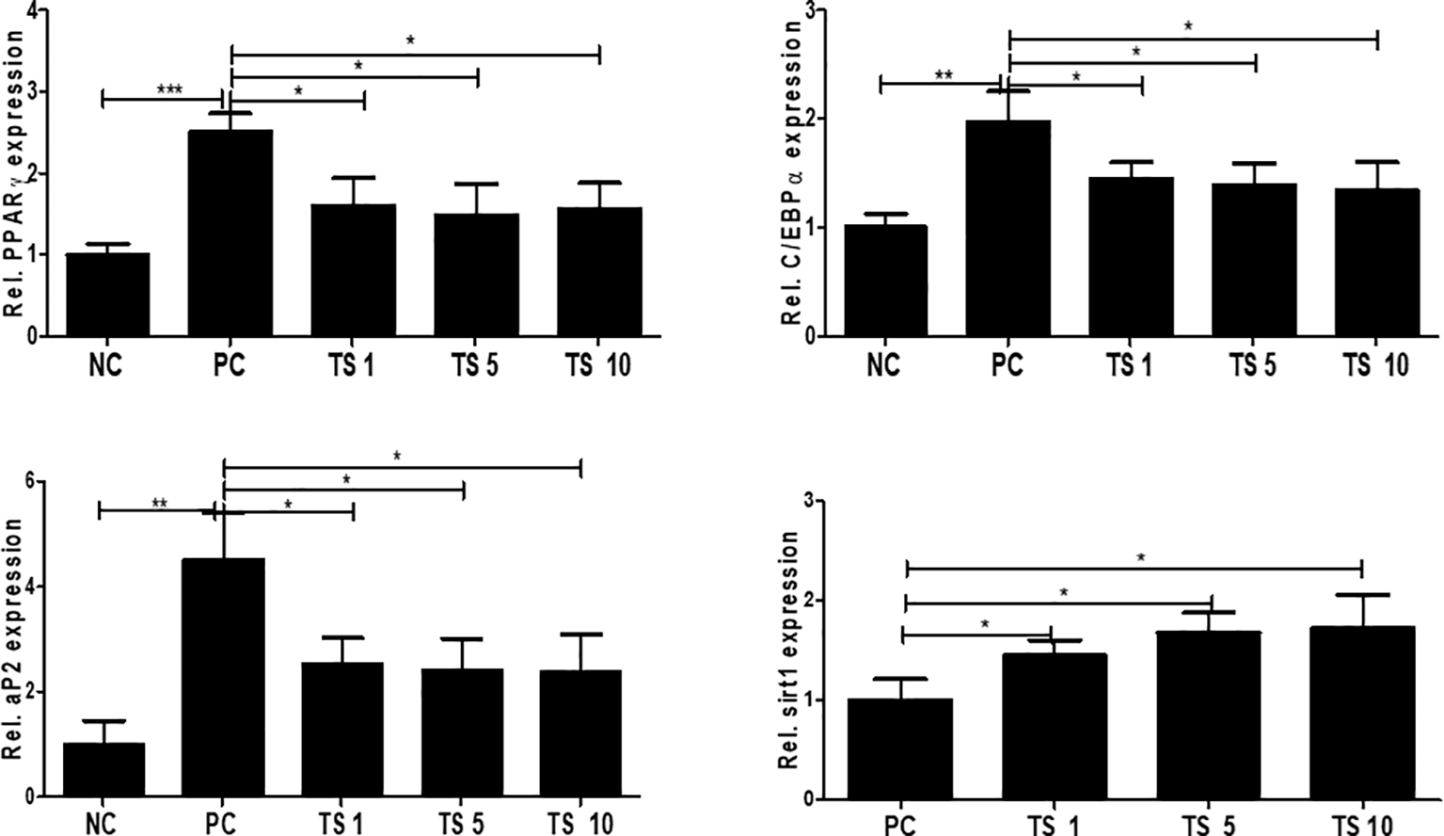
The effect of *T. spiralis* total lysates on adipogenic genes mRNA and protein expression. 3T3-L1 adipocytes were treated with 1, 5, or 10 μg *T. spiralis* total lysates during differentiation. RNA and proteins were isolated from the cells at the end-stage of differentiation. Relative mRNA expression confirmed the expression of adipogenic genes. The results show that *T. spiralis* total lysates treatment clearly decreased the expression of PPARγ, C/EBPα, and aP2 mRNA. Bars represent the means ± SD from three independent experiments. (*; p < 0. 05, ** p < 0.01 ***; p < 0.001*** n = 4; samples per group, two independent experiments). NC, negative control, undifferentiation; PC, positive control, differentiation; TS 1, treatment of *T. spiralis* total lysates 1 µg; TS 5, treatment of *T. spiralis* total lysates 5 µg; TS 10, treatment of *T. spiralis* total lysates 10 µg.

**Figure 11:**
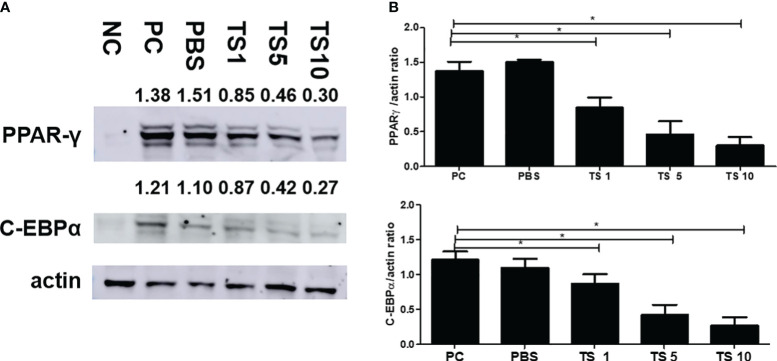
Effect of *T. spiralis* total lysates on adipogenic factors. The cells were seeded at a density of 8 × 104 cells/well in a 6-well plate. Differentiation was induced with or without *T. spiralis* total lysate for up to 10 days. **(A)** Steady-state levels of PPARγ, C/EBPα and actin were evaluated by western blot analysis. Results are expressed as the mean ± SEM. ∗p < 0.05. **(B)** Graphs show the results of three separate experiments. NC, negative control, undifferentiation; PC, positive control, differentiation; PBS, treatment of PBS; TS 1, treatment of *T. spiralis* total lysates 1 µg; TS 5, treatment of *T. spiralis* total lysates 5 µg; TS 10, treatment of *T. spiralis* total lysates 10 µg.

To investigate the molecular mechanisms underlying the antiadipogenic effect of *T. spiralis* total lysates, we evaluated mRNA expression levels of C/EBPα, and PPARγ as key regulatory factors of adipogenesis (Jackson et al., 2017). As expected, after 10 days, *T. spiralis* total lysates treatment during cellular differentiation significantly decreased expression of PPARγ and C/EBPα, compared to control cells (**Figure 10**). In particular, we examined PPARγ and C/EBPα protein levels in completely differentiated adipocytes treated with *T. spiralis* total lysates. PPARγ and C/EBPα levels were significantly reduced by *T. spiralis* total lysates compared to control cells in a concentration dependent manner (**Figure 11**) (p < 0.05). In addition, inhibition in adipocyte differentiation was also evaluated by measuring aP2 levels, which are specific markers of adipose tissue differentiation (Ertunc et al., 2015; Palhinha et al., 2019). *T. spiralis* total lysates significantly reduced the levels of aP2, confirming the effect of *T. spiralis* total lysates as an antiadipogenic compound (**Figure 10**). Particularly, Sirt1 inactivation or deletion induces metabolic dysfunction and an increase in adipose tissue (Chalkiadaki and Guarente, 2012). Accordingly, a significant improvement in Sirt1 expression was present following *T. spiralis* total lysates (p < 0.05), compared to untreated cells, suggesting that the antiadipogenic effect of *T. spiralis* total lysates may be mediated by the Sirt1/PPARγ pathway. Taken together, these results highlight the role of *T. spiralis* total lysates in reducing adipocyte differentiation, which may markedly reduce the accumulation of adipose cell mass.

## Discussion

Parasites have evolved to regulate the host immune system and tissue repair responses to induce survival by modulating inflammation that would otherwise induce worm expulsion (Gomes et al., 2016). Parasitic infections generally drive a type 2 immune response, thereby maintaining an anti-inflammatory immune microenvironment and improving host metabolic diseases including obesity (van der Zande et al., 2019). Our research group has focused on the infective immunity of *T. spiralis* for a long time. We previously reported the accumulation of immunomodulatory cells in the host following infection with this parasite (Kang et al., 2012; Kang et al., 2014; Kang et al., 2019; Kang and Yu, 2021; Kang et al., 2021; Park et al., 2022). Moreover, parasite-derived excretory-secretory products can suppress pro-inflammatory responses *in vivo* and *in vitro* (34, 36). Recently, we found that parasitic infections ameliorated diet-induced obesity in mice (Kang et al., 2021).

The relationship between the metabolism and immune response has become an important issue. Adipose tissue is a major immune organ in metabolism. It has been shown that changes in the metabolic state of an individual later result in changes in the immune balance. Obesity mainly weaken Th2 responses, while strengthening type 1 inflammatory responses. Whereas, hunger and malnutrition result in a type 2 biased immune response (Schmidt et al., 2022).

Th2 cells are beneficial to the homeostatic environment of adipose tissue in lean individuals. A question may be raised as to whether the induction of Th2 cells during obesity has an effect in improving obesity. Several studies reveals that helminth infections to induce a Th2 and regulatory condition that may impact the seriousness of irrelevant inflammation and illness (Ryan et al., 2020).

Previous studies, including ours, have shown that parasitic infection changes the differentiation of adipose tissues and metabolism in mice (Lu et al., 2020). Based on our previous data from murine models, *T. spiralis* infection significantly induces Th2 immune response and M2 macrophage polarization, which ameliorates diet-induced obesity. Several studies have revealed that infection of diet-induced obese mice with *Heligmosomoides polygyrus Schistosoma mansoni* and *Nippostrongylus brasiliensis* alleviates whole-body glucose tolerance and insulin sensitivity (Weng et al., 2007; Yang et al., 2013; Su et al., 2018). These results show an inverse relation between helminth infection and the incidence of metabolic syndrome (Wiria et al., 2012).

This study aimed to determine the effect of *T. spiralis* total lysate treatment on diet-induced obesity (*in vivo*) and adipocyte differentiation (*in vitro*). First, we investigated the effects of *T. spiralis* total lysates treatment in diet-induced obese mice. Secondly, we investigated whether the *in vitro* anti-obesity effects were retained. The present study investigated the antiadipogenic effects underlying the action of *T. spiralis* total lysate on 3T3-L1 adipocytes.

Our results showed that *T. spiralis* total lysates treatment significantly promotes M2 macrophage polarization and inhibits the proinflammatory cytokine as IL-1β and TNF-α.

Our results are consistent with prior reports that helminth infection alleviate obesity (Weng et al., 2007; Yang et al., 2013; Su et al., 2018; Yao et al., 2022) T cell derived cytokines play a pivotal role for generation of proinflammatory M1 macrophages during obesity. Therefore, the decreased production of IL-4 and IL-10 within the adipose tissue results in a relative decrease of anti- inflammatory M2 macrophages. Whereas, the increase of Th1 cells promotes M1 macrophages (Winer et al., 2009). Our results showed that there was no significant increase of IL-4 and IL-10, but *T. spiralis* total lysate treatment significantly inhibited the production of Th1 cells. Leptin is an adipokine produced primarily by adipocytes, although it is produced by various cells, such as immune cells. Therefore, increasing adiposity result in increased systemic concentrations of leptin. Studies provided evidence that leptin has been identified as a critical immune modulator in an obesity-associated inflammation by promoting pro-inflammatory immune response (Kiernan and MacIver, 2020; Perakakis et al., 2021). Our results showed that *T. spiralis* total lysate treatment reduced the amount of fat mass and the expression level of leptin in a white adipose tissues. Preadipocytes differentiate into mature adipocytes. Hyperplasia and hypertrophy of these cells can result in an increase in adipose tissue mass (Kim et al., 2013; Chen et al., 2014). During adipocyte differentiation, intracellular lipid droplets build up in mature adipocytes (Kim et al., 2013). Intracellular lipid content was measured using spectrophotometry and lipid accumulation was evaluated using Oil Red O staining. The results demonstrate that *T. spiralis* total lysates suppress the differentiation of 3T3-L1 adipocytes. Specifically, 3T3-L1 adipocytes treated with *T. spiralis* total lysate showed decreased lipid accumulation. Adipocyte differentiation is related to mainly by a cascade reaction entailing transcription factors such as C/EBPα and PPARγ (Farmer, 2006). The differentiation of preadipocytes into mature adipocytes is divided into early and late stages.

In the early stages, the transcription factors C/EBPβ and C/EBPδ are caused by specific hormones. Subsequently, PPARγ and C/EBPα are caused by these factors (Cao et al., 1991). PPARγ and C/EBPα then induce adipogenesis by modulating in a positive manner (Cao et al., 1991; Kim et al., 2018). Consistent with the data from the Oil Red O staining, the results of the present study revealed that *T. spiralis* total lysates significantly decreased the expression of PPARγ and C/EBPα mRNA and proteins, which are pivotal adipogenic transcription factors related to the final stage of adipocyte differentiation. Additionally, the expression levels of adipogenic marker genes including aP2 were decreased by *T. spiralis* total lysate treatment. These findings suggest that *T. spiralis* lysates reduce adipogenesis by regulating C/EBPα and PPARγ pathways. Further, Sirt1 is a major modulator of adipogenesis and lipid metabolism; it belongs to the 7 sirtuins, which are class III histone deacetylases that regulate senescent process and metabolic homeostasis (Li and Kazgan, 2011). Further, Sirt1 weakens adipogenesis by impairing PPARγ expression (Picard et al., 2004).

We used two methods for *T. spiralis* total lysate treatment: IO and IP administration. However, no significant differences were observed between the two administration methods. There was a difference in the absorption time between the two methods, but there was no significant difference in the anti-obesity effect.

In conclusion, the present study demonstrated that changes in immune cell composition caused by *T. spiralis* total lysate treatment improved glucose tolerance in mice fed a high-fat diet. *T. spiralis* total lysates inhibit the expression of adipogenic marker genes and lipid accumulation through down-regulation of C/EBPα and PPARγ. Further studies are required to identify specific molecules involved in the prevention and treatment of obesity through such pathways. Taken together, results from our study raise the interesting possibility of using parasite lysates as effective and novel therapeutics in the treatment of obesity.

## Data availability statement

The original contributions presented in the study are included in the article/supplementary materials, further inquiries can be directed to the corresponding author/s.

## Ethics statement

The animal study was approved by Pusan National University Animal Care and Use Committee. The study was conducted in accordance with the local legislation and institutional requirements.

## Author contributions

HY: Conceptualization, Funding acquisition, Project administration, Resources, Supervision, Writing – review & editing. SK: Data curation, Formal analysis, Investigation, Methodology, Validation, Visualization, Writing – original draft.
